# Synchronizing an aging brain: can entraining circadian clocks by food slow Alzheimer’s disease?

**DOI:** 10.3389/fnagi.2014.00234

**Published:** 2014-09-01

**Authors:** Brianne A. Kent

**Affiliations:** Department of Psychology, University of CambridgeCambridge, UK

**Keywords:** circadian rhythms, Alzheimer’s disease, food-entrainment, ghrelin, aging

## Abstract

Alzheimer’s disease (AD) is a global epidemic. Unfortunately, we are still without effective treatments or a cure for this disease, which is having devastating consequences for patients, their families, and societies around the world. Until effective treatments are developed, promoting overall health may hold potential for delaying the onset or preventing neurodegenerative diseases such as AD. In particular, chronobiological concepts may provide a useful framework for identifying the earliest signs of age-related disease as well as inexpensive and noninvasive methods for promoting health. It is well reported that AD is associated with disrupted circadian functioning to a greater extent than normal aging. However, it is unclear if the central circadian clock (i.e., the suprachiasmatic nucleus) is dysfunctioning, or whether the synchrony between the central and peripheral clocks that control behavior and metabolic processes are becoming uncoupled. Desynchrony of rhythms can negatively affect health, increasing morbidity and mortality in both animal models and humans. If the uncoupling of rhythms is contributing to AD progression or exacerbating symptoms, then it may be possible to draw from the food-entrainment literature to identify mechanisms for re-synchronizing rhythms to improve overall health and reduce the severity of symptoms. The following review will briefly summarize the circadian system, its potential role in AD, and propose using a feeding-related neuropeptide, such as ghrelin, to synchronize uncoupled rhythms. Synchronizing rhythms may be an inexpensive way to promote healthy aging and delay the onset of neurodegenerative disease such as AD.

## Alzheimer’s disease

Alzheimer’s disease (AD) is a progressive neurodegenerative disorder associated with severe amnesia and a variety of other cognitive and behavioral impairments. AD is the most common form of dementia, which is estimated to be affecting more than 44.4 million people worldwide, making dementia a global epidemic and one of the greatest public health challenges of the 21st century (Prince et al., 2013). There is an urgent need to develop effective interventions to prevent, delay the onset, and slow the progression of AD. Unfortunately, we are currently without beneficial treatments or a cure for this devastating disease.

The majority of research efforts are focused on identifying specific mechanisms of disease and developing compounds to target the underlying disease pathology. However, finding ways to promote overall health is arguably, just as important. For 60 to 70 years our body’s natural defenses are able to ward off disease progression. Once we reach an unknown threshold, our repair and clearance mechanisms- which slow with age- are no longer able to counteract the damage to our cells (Martinez-Vicente et al., 2005; Martinez-Vicente and Cuervo, 2007; Rattan, 2010). This slowing then results in intracellular accumulation of misfolded proteins that form toxic multimetric complexes. The progressive accumulation of toxic structures inside cells is one of the main molecular characteristics of aging and is associated with most neurodegenerative diseases, including AD (Martinez-Vicente and Cuervo, 2007; Rattan, 2010).

We need to find ways to shift this delicately poised boundary to slow the aging process, and postpone when age-related diseases become problematic. Even relatively small changes, that delay the onset and spread of AD and other age-related conditions, have the potential to make a big impact on the quality of life of patients and reduce the costs to society.

The slow progression of AD emphasizes the need to focus on health promotion and prevention (Gandy and DeKosky, 2013). In autosomal dominant AD, a longitudinal study showed that patients had pathophysiological changes in cerebrospinal fluid biochemical markers up to 25 years prior to symptom onset (Bateman et al., 2012). It is unclear whether the same timeline applies to patients who develop sporadic AD, but it is hypothesized that sporadic AD also takes over a decade to develop before the onset of symptoms (Gandy and DeKosky, 2013; Jack et al., 2013).

AD pathology does not disrupt memory functioning in isolation, there are wide-spread effects throughout the body affecting physiological processes such appetite, sleep patterns, emotional regulation, and sense of smell (Witting et al., 1990; Mok et al., 2004; Tabert et al., 2005; Cai et al., 2012). There is evidence to suggest that some of these symptoms, such as circadian dysfunction and weight loss, may preface the clinical onset of memory and executive functioning deficits (Buchman et al., 2005; Knopman et al., 2007; Coogan et al., 2013).

Chronobiological concepts may provide a useful framework for understanding the link between the early changes in feeding behavior and sleep patterns, and the development of age-related disease such as AD. Disturbances of daily sleep-wake behaviors are common in AD, and are a leading cause for institutional care of patients (Bianchetti et al., 1995), thus there is great potential for identifying inexpensive and effective interventions by studying the circadian system in AD.

Understanding the physiological changes that contribute to AD and finding ways to promote overall health requires a systems level approach. The following review will provide a brief introduction to the circadian system, its potential role in AD, and propose adopting what we know from the food-entrainment literature to evaluate the potential for using a feeding-related neuropeptide, such as ghrelin, to synchronize uncoupled rhythms. Synchronizing rhythms may be an inexpensive way to promote healthy aging and delay the onset of neurodegenerative disease such as AD.

## Circadian rhythms

Circadian rhythms refer to a complex and distributed system imposing a temporal architecture on physiology and behavior. These rhythms are generated by hierarchically organized central and peripheral oscillators entrained (i.e., synchronized) by periodic stimuli in the external environment or within the body.

Biological clocks maintain synchrony between cycles in the environment and our physiological processes and behavior, to ensure that daily peaks in energy metabolism, gastrointestinal tract motility, cardiovascular activity, endocrine secretion, body temperature, and cognitive processes occur at optimal times of the day (Panda et al., 2002; Reppert and Weaver, 2002; Kalsbeek et al., 2006; Schmidt et al., 2007; Gerstner and Yin, 2010; Waterhouse, 2010; Huang et al., 2011; Konturek et al., 2011). Coordinating internal processes with the external environment optimizes energy expenditure and may also provide an evolutionary or survival advantage by ensuring activities are performed at appropriate times of the day to find food and mating partners, and avoid predators (Panda et al., 2002; Schibler et al., 2003; Woelfle et al., 2004). Further selective advantage of the circadian clocks may also come from temporally separating incompatible intracellular processes, ensuring that specific proteins are expressed at ideal time points (Rey and Reddy, 2013).

The core clock machinery is self-sustaining and has an endogenous period that approximates 24 h. A zeitgeber (i.e., entraining cue) is required to synchronize rhythms to match the 24 h day and keep internal processes aligned with the external environment. Although several parameters change in the daily environment, such as light, temperature, and humidity, the daily light-dark cycle is considered the dominant zeitgeber.

**Figure 1:**
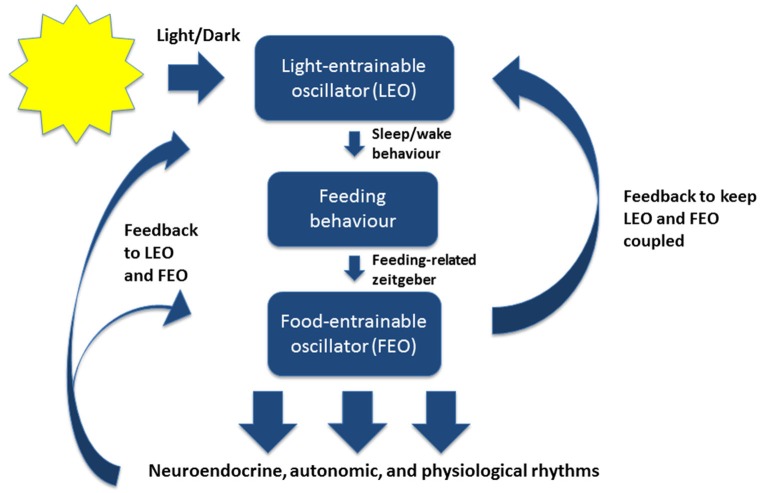
**A simplified model of the mammalian circadian system under normal conditions**. The light-entrainable oscillator (LEO) is located in the suprachiasmatic nucleus (SCN) of the hypothalamus. The LEO is directly entrained by light-dark cycles in the environment through intrinsically photoreceptive retinal ganglion cells, and generates circadian rest-activity rhythms as well as other rhythms that entrain to the environmental light-dark cycles. The LEO-driven activity rhythms then influence the timing of feeding behavior, which entrains the food-entrainable oscillator (FEO). FEO then drives several neuroendocrine, autonomic, and physiological rhythms throughout the brain and peripheral nervous system. The FEO also sends feedback to the LEO to remain coupled. Under certain conditions when the LEO is dysfunctional, or under conditions of constant darkness, the FEO can drive rest-activity rhythms and other rhythms normally entrained by light.

In mammals, the master circadian pacemaker is the suprachiasmatic nucleus (SCN) of the hypothalamus, which is located dorsal to the optic chiasm (Weaver, 1998; Reppert and Weaver, 2002; Karatsoreos and Silver, 2007; Welsh et al., 2010). The SCN is directly entrained by light-dark cycles in the environment through intrinsically photoreceptive retinal ganglion cells (Figure 1; Berson et al., 2002).

Outside the SCN there are circadian oscillators in peripheral organs and tissues, regulating local cycles of gene expression, physiology, and metabolic rhythms (Yamazaki et al., 2000; Guilding and Piggins, 2007; Kornmann et al., 2007; Dibner et al., 2010). Rhythmic expression of clock-controlled genes (e.g., PER1,2, CRY1,2, CLOCK, BMAL1), which are produced by a series of interlocking transcriptional feedback/feedforward loops, drive physiological and behavioral rhythms (Reppert and Weaver, 2002). Through neural, hormonal, and behavioral outputs the SCN directly and indirectly coordinates this multioscillatory system, synchronizing rhythms throughout the central and peripheral nervous systems (Dibner et al., 2010). By having a master pacemaker (i.e., SCN) to align circadian gene oscillations within peripheral tissues, oscillators throughout the body remain coupled together, synchronizing cellular and physiological processes with daily cycles in the external environment.

When the SCN is unable to entrain to light-dark cycles, such as when the SCN is experimentally ablated or an animal is in conditions of constant darkness, daylight no longer acts as a zeitgeber and circadian organization gradually becomes out of sync with the environment (i.e., free running rhythms persist). However, under certain conditions, another powerful zeitgeber- food- can restore, synchronize, and propagate circadian rhythmicity (Boulos and Terman, 1980; Stephan, 2002).

## Food-entrainment

The process whereby food/mealtime acts as the stimulus for synchronizing the circadian system is referred to as “food entrainment”. There is strong evidence that an SCN-independent “food-entrainable” oscillator exists, possessing the canonical properties of a circadian clock, although its location has not yet been identified (Boulos and Terman, 1980; Mistlberger, 1994, 2009; Stephan, 2002; Patton and Mistlberger, 2013).

If food is restricted to one or two daily mealtimes, then within a few days rodents begin to show food anticipatory activity (FAA), which is a higher level of activity (e.g., wheel running or exploration of their home cage) during the few hours leading up to the daily meal (Boulos and Terman, 1980; Mistlberger, 1994; Stephan, 2002). FAA is thought to represent food seeking behavior. Scheduled feeding can entrain the entire circadian system under conditions of constant darkness, or can dissociate peripheral rhythms from the central SCN-driven rhythms under normal conditions with a dominate light-dark cycle (Damiola et al., 2000).

It is not yet known what the specific mechanism is by which mealtime exerts its entraining effect on the circadian system. There may be multiple food-related stimuli that are capable as functioning as zeitgebers, entraining both central and peripheral oscillators. For example, metabolic hormones (e.g., ghrelin, corticosterone, leptin, insulin, and glucagon) exhibit daily rhythms of synthesis and secretion that are synchronized by meal time (Patton and Mistlberger, 2013). These hormones may act as signals by which oscillators in peripheral organs are coupled to daily mealtime (Dibner et al., 2010).

Importantly, because particular feeding schedules can shift the timing of clock genes and rhythms in peripheral organs, dissociating peripheral oscillators from the SCN, it suggests that feeding behavior could be the mediating factor by which SCN coordinates peripheral physiological rhythms (Figure 1; Boulos and Terman, 1980; Damiola et al., 2000; Dibner et al., 2010). In other words, feeding behavior may keep central and peripheral rhythms coupled together.

Although the specific mechanism by which food/mealtime exert the powerful synchronizing effects on peripheral rhythms, remains unknown, once it is identified, targeted interventions to help synchronize circadian rhythms can be developed. Synchronizing rhythms may be beneficial for preventing or delaying age-related conditions if rhythms become uncoupled.

## Harmful effects of disrupted circadian rhythms

The importance of keeping the circadian system synchronized is illustrated by the harmful outcomes of disrupted rhythms. It is well reported that circadian dysfunction can have dramatic effects on health in both animal models and humans. Disturbances in the circadian system can have negative effects on cognition, immune functioning, metabolic processes, and the cardiovascular system, resulting in fatigue, disorientation, insomnia, altered hormone profiles, higher morbidity, and higher mortality in humans and animal models (Penev et al., 1998; Fu et al., 2002; Filipski et al., 2003; Davis and Mirick, 2006; Fujino et al., 2006; Kubo et al., 2006; Coogan and Wyse, 2008; Anea et al., 2009; Gibson et al., 2009; Gery and Koeffler, 2010; Jung-Hynes et al., 2010; Coogan et al., 2013).

In humans, the experience of jet-lag or shift work represents a state when the circadian system is out of sync with the environment, and is associated with damaging effects on health. Shift-workers are at a higher risk of harmful conditions such as metabolic syndrome, obesity, cancer, and diabetes, which may partly result from the disrupted rhythmicity of meals and sleep (Bass and Takahashi, 2010).

In animals, disrupted circadian rhythms also result in suboptimal health (Filipski et al., 2003; Anea et al., 2009; Wang et al., 2010). For example, in aged mice, phase shifting (analogous to jetlag) is associated with higher mortality (Davidson et al., 2006). Similarly, continuous reversal of the light-dark cycle, decreases survival in cardiomyopathic hamsters (Penev et al., 1998).

What jet-lag, shift work, and phase shifting have in common, is that underlying rhythms become uncoupled and out of sync. Uncoupling metabolic process from circadian drives may reduce efficiency and cause imbalances in physiological activities. A similar process may underlie age-related changes and contribute to neurodegenerative disease.

## Circadian rhythms and neurodegenerative disease

It is well documented that circadian rhythms become disorganized and lose amplitude during healthy aging and neurodegenerative disease (Witting et al., 1994; Hofman and Swaab, 2006; Kondratova and Kondratov, 2012). In rodent models, there are age-related changes in the rhythms of parameters such as body temperature, activity-wakefulness, locomotor activity, and drinking behavior (Weinart, 2000; Kolker et al., 2003). Aged animals given fetal SCN implants have increased longevity, suggesting that age-related impairments may be due to changes in the SCN (Hurd and Ralph, 1998).

In humans, similar age-related changes in melatonin secretion, body temperature rhythms, and sleep-wake cycles are also experienced (Duffy et al., 2002; Yoon et al., 2003; Hofman and Swaab, 2006). These circadian disruptions are particularly common in patients with AD, who often complain of disrupted sleep patterns as well as agitation, restlessness, wandering, and verbal outbursts during the late afternoon or early evening, referred to as “sundowning” and occurs in an estimated 13–66% of AD patients (Volicer et al., 2001). Along with these behavioral symtpoms, dementia patients also show disordered melatonin rhythms (Mishima et al., 1999).

Importantly, the results of a large prospective study suggested that the age-related changes in circadian activity patterns, predicted subsequent AD or mild cognitive impairment (Tranah et al., 2011). Thus, impaired rhythms may be a preclinical biomarker of disease.

Monitoring skin temperature is useful for assessing circadian rhythmicity at the gross level. One study showed that proximal skin temperature but not distal skin temperature was higher during the day in AD patients compared with healthy elderly subjects (Most et al., 2012). Higher proximal skin temperature was associated with daytime sleepiness, which could exacerbate cognitive symptoms.

Actigraphy, a noninvasive method for monitoring rest/activity cycles, is another technique for evaluating rhythmicity in AD patients (Witting et al., 1990; Satlin et al., 1992; van Someren et al., 1996; Ancoli-Israel et al., 2003a; Hatfield et al., 2004). Daily activity-rest rhythms of demented patients exhibit increased fragmentation, a loss of amplitude, and higher nighttime activity (van Someren et al., 1996; Hatfield et al., 2004). These rest-activity disturbances may be correlated with the severity of dementia (Witting et al., 1990). AD patients also show reduced scale invariance of activity fluctuations (Hu et al., 2009). This parameter is found to be dependent upon the SCN in rodents (Hu et al., 2007), suggesting that the changes in scale-invariant locomotor patterns in AD patients may reflect SCN dysfunction.

Post mortem studies have revealed neuropathological changes in the SCN of healthy elderly and AD patients (reviewed by Hofman and Swaab, 2006). Although there have been some inconsistencies, possible pathological changes in the SCN of AD patients include decreased vasopressin-, neurotensin- and melatonin-expressing neurons, increased astrocyte to neuron ratio, neuronal loss in the SCN, and an overall decrease in SCN volume (Swaab et al., 1985; Stopa et al., 1999; Liu et al., 2000; Wu et al., 2007; Harper et al., 2008). Neurodegeneration of SCN in AD patients is correlated with the magnitude of circadian rhythm impairment in core body temperature (Harper et al., 2008).

AD patients also show desynchrony in rhythmic expression of circadian clock genes in the oscillation between cingulate cortex, pineal gland and the bed nucleus of the stria terminalis (BNST; Cermakian et al., 2011), suggesting that the circadian oscillators outside the SCN become uncoupled in AD patients. Hatfield et al. (2004) came to a similar conclusion when they compared activity/rest and cortisol rhythms in AD patients and concluded that the loss of circadian control of rest/activity was not a result of a global circadian disruption.

There are a few potential pathways by which circadian rhythms could be affected in AD (for a review see Coogan et al., 2013). The input from photic and non-photic zeitgebers may become weakened due to age-related changes in physiology and behavior. For example, with increasing age people often experience changes in eye functioning and have routines with less physical exercise, both of which could reduce input into the circadian system. Older age and retirement often result in more sporadic scheduling of activities such as mealtime, which can play an important role in synchronizing central and peripheral rhythms in animal models. The reduced input from these changes in physiology and behavior may then be compounded by changes in SCN and weakened internal feedback mechanisms within the body. If the resulting SCN output is weak, then pineal activity, hippocampal functioning, and the HPA axis are affected.

Given the considerable evidence of circadian dysfunction in AD patients, chronotherapeutics targeting circadian abnormalities have been used in attempts to reset the clock (Coogan et al., 2013). The most marked circadian abnormality in AD patients is reduced amplitude of rhythms and rhythm fragmentation, suggesting that interventions should be aimed at strengthening zeitgebers instead of phase-resetting. Changes to the patient’s environment (e.g., light therapy), behavioral routines (e.g., exercise), and circulating hormone levels (e.g., melatonin supplementation), have all been attempted to strengthen rhythmicity.

Evening and/or morning light therapy have been shown to help stabilize rhythms and improve sleep (Satlin et al., 1992; Mishima et al., 1994; Yamadera et al., 2000; Ancoli-Israel et al., 2003b). One study found that in institutionalized patients, light therapy was only minimally beneficial at stabilizing circadian phase, but was more beneficial when combined with melatonin treatment (Dowling et al., 2008). A double-blind study showed that melatonin treatment improved cognition, decreased nocturnal activity, and increased nocturnal sleep in AD patients (Asayama et al., 2003), although larger follow-up studies have showed no benefits of melatonin treatment (Singer et al., 2003; Gehrman et al., 2009).

Along with promoting health, regulating circadian rhythmicity may also affect wellbeing. Elderly demented women with higher daytime activity levels and lower nocturnal activity (i.e., consolidated, nonfragmented sleep/wake cycles) showed higher levels of wellbeing compared to elderly women with more disrupted sleep-patterns (Carvalho-Bos et al., 2007).

Given the difficulty of assessing core circadian processes in patients, animal models are useful for elucidating circadian alterations. Rats injected with transgenic cells overexpressing β/A4 amyloid into the SCN, displayed disrupted locomotor rhythms indicative of deterioration of circadian regulation (Tate et al., 1992). Similarly, hamsters injected with β-amyloid 25–35 into the SCN, showed phase-advanced and less consistent diurnal activity rhythms (Furio et al., 2002). These effects were attenuated by melatonin administration.

Some transgenic mouse models of AD such as APP23, Tg2576, and 3xTg display disturbances in circadian activity rhythms (Van Dam et al., 2003; Vloeberghs et al., 2004; Sterniczuk et al., 2010). The 3xTg AD mouse model shows abnormalities in circadian rhythmicity that precede AD pathology (Sterniczuk et al., 2010). In contrast, no circadian abnormalities in sleep-wake behavior were reported in the AβPPswe/PSEN1A246E or SPPswe/PS1dE9 transgenic mouse of AD (Jyoti et al., 2010; Otalora et al., 2012).

To summarize, disrupted circadian rhythms are associated with higher morbidity and higher mortality in humans and animal models. With increasing age, rhythmicity becomes more irregular and these changes are more extreme for AD patients and may precede the cognitive impairment associated with AD. Light therapy has had some success at regulating circadian rhythmicity and improving symptoms in AD patients.

## Uncoupled hypothesis: using food-entrainment to synchronize rhythms

It is largely assumed that the circadian disruptions associated with age and neurodegenerative diseases, such as AD, are caused from the dampening of central SCN-driven rhythms. However, another possibility is that the disrupted patterns in behavior and physiological rhythms are due to the central rhythms becoming uncoupled from and out of sync with peripheral rhythms, and not from disruptions in central clock functioning.

Chen et al. (2014) used a transgenic *Drosophila* model, to provide the first evidence that progressive circadian deficits analogous to those experienced by AD patients were not due to an arrhythmic internal timekeeping mechanism, but rather due to a disruption in the communication between a central timekeeping mechanism and peripheral rhythms. The Aβ expression in the *Drosophila* model resulted in age-related disturbances in circadian rhythms, causing sporadic sleep-wake behaviors. Despite the behavioral irregularities, the central timekeeping mechanism of this *Drosophila* model appeared intact. The behavioral arrhythmia was not due to a failing central clock.

If these findings translate to AD patients, then it would suggest that the SCN would stay entrained to daylight while the peripheral rhythms affecting behavior become out of sync. If circadian behavioral abnormalities in patients are not caused by a loss of SCN function, then it could have potential implications for the treatment and prevention of AD.

For example, if in AD patients, the SCN is intact but central and peripheral rhythms become uncoupled, then, because feeding plays a dominate role in synchronizing central and peripheral rhythms, it may be possible to draw on the food-entrainment literature for insights into how we can bring physiological processes back in sync.

Maywood et al. (2010) used a food-entrainment paradigm to restore circadian disturbances in behavior and peripheral metabolic processes in the R6/2 mouse model of Huntington’s disease. This demonstrates that it is possible to regularize circadian behavioral and metabolic disturbances using food-entrainment in a mouse model of neurodegenerative disease.

Food-entrainment paradigms would be challenging to introduce as therapies for human patients, but there may be a way to pharmacologically induce synchrony between central and peripheral rhythms using a feeding-related peptide such as ghrelin.

## Ghrelin: circadian system

The metabolic hormone, ghrelin, may be one promising candidate signal that could be particularly useful for synchronizing rhythms and preventing age-related memory loss in AD.

Ghrelin is an orexigenic hormone synthesized by oxyntic cells in the stomach and by neurons in the medial and lateral hypothalamic nuclei (Kojima et al., 1999; Cowley et al., 2003). It is a peripheral and central hormone directly implicated in feeding related activity, and an endogenous ligand for the growth hormone secratague receptor (GHS-R1a; Kojima et al., 1999).

Ghrelin acts in the pituitary and hypothalamus to stimulate growth hormone secretion, energy homeostasis, appetite, wakefulness, weight gain, and adrenocorticotropic hormone and cortisol release (Kojima et al., 1999; Wren et al., 2000; Tschöp et al., 2000; Kojima and Kangawa, 2005; Szentirmai et al., 2006; Chen et al., 2009; Castañeda et al., 2010). Ghrelin stimulates feeding (Nakazato et al., 2001; Toshinai et al., 2006), and circulating levels rise prior to mealtime in both rodents (Bodosi et al., 2004; Drazen et al., 2006; LeSauter et al., 2009) and humans (Cummings et al., 2001; Frecka and Mattes, 2008).

In addition to regulating appetite, there is evidence that ghrelin also plays a role in the circadian system by directly entraining circadian clocks that drive behavior or indirectly by stimulating appetite and activity. Ghrelin positive immunoreactive neurons are found in brain regions involved for circadian timing such as the paraventricular, dorsomedial, ventromedial, and arcuate nuclei, and may affect SCN processes via the ventromedial arcuate nucleus (Cowley et al., 2003; Yi et al., 2006). Under restricted feeding conditions, the circadian system is sensitive to ghrelin and other feeding-related neuropeptides (Yannielli et al., 2007). Additionally, peripherally administered ghrelin has been shown to modulate SCN activity in rats and mice and attenuate light-induced phase delay in mice (Yi et al., 2008).

However, there is mixed evidence as to whether ghrelin can act a zeitgeber. Two studies show that ghrelin receptor knock out mice fail to anticipate a daily meal (LeSauter et al., 2009; Davis et al., 2011; Verhagen et al., 2011), however other studies have shown that ghrelin ligand or receptor knock-out mice do continue to show FAA (Blum et al., 2009; Szentirmai et al., 2010; Gunapala et al., 2011).

Overall, the evidence suggests that ghrelin is not the unidentified “food-entrainable oscillator” but rather that it exerts effects on the circadian system by acting downstream from or in parallel with a food entrainable oscillator (Patton and Mistlberger, 2013). Even though ghrelin may not be necessary for anticipatory activity it may still promote its expression by strengthening the zeitgeber.

Another useful paradigm for studying food-entrainment, which may be more translatable to humans, is examining the effects of a daily palatable snack or meal on the circadian system of animals that are not food-restricted. Without caloric restriction, a palatable snack (e.g., chocolate) can engender food anticipatory behavior (Mistlberger and Rusak, 1987; Mendoza et al., 2005; Hsu et al., 2010).

Ghrelin has been examined as a modulator factor in palatable meal anticipation with mixed results. One study found that plasma ghrelin levels correlated with locomotor activity counts during the 3 h prior to the chocolate snack, and that injected ghrelin increased anticipatory activity (Merkestein et al., 2012). However, another study found no increase in ghrelin prior to a daily chocolate snack (Dailey et al., 2012).

Overall, evidence suggests that ghrelin could be involved in the mechanism by which mealtime acts as a zeitgeber. However, there is not enough evidence to draw conclusions about whether ghrelin could be useful for synchronizing central and peripheral rhythms in the elderly at risk for AD. It remains an empirical question whether appropriately timed daily administration of ghrelin could help to amplify food-entrainment to daily scheduled meals in the elderly. If the uncoupling of rhythms contributes to age-related neurodegenerative disease, then combining ghrelin treatment with scheduled mealtimes may help synchronize rhythms. It is possible that bringing out-of-sync rhythms back in sync could promote healthy aging, delay the onset of neurodegenerative disease, and may even help reduce symptoms and improve wellbeing.

## Ghrelin: memory and Alzheimer’s disease

Although the potential of ghrelin to entrain the circadian system of elderly at risk for AD remains to be confirmed, recent evidence suggests that administering ghrelin may have other potential benefits as well. Extra-hypothalamic actions of ghrelin have been identified, including pro- cognitive, antidepressant, (anti-)anxiogenic, and neuroprotective properties (Asakawa et al., 2001; Kanehisa et al., 2006; Lutter et al., 2008; Andrews, 2011; Frago et al., 2011; Steiger et al., 2011). Consistently, intracranial infusions and systemic ghrelin treatments have beneficial mnemonic effects (Carlini et al., 2002, 2004, 2007, 2008, 2010a,b; Diano et al., 2006; Atcha et al., 2009; Tóth et al., 2010; Chen et al., 2011; Chen, 2012), affect measures of hippocampal synaptic plasticity (Diano et al., 2006; Carlini et al., 2010b; Chen et al., 2011), and promote hippocampal neurogenesis (Moon et al., 2009; Chen, 2012).

Carlini et al. (2002) were the first to demonstrate that ghrelin treatment can improve memory retention. The researchers injected the peptide intracerebroventricularly (ICV) in rats and found that the treatment improved memory in a dose-dependent manner, as measured by latency time in a step-down behavioral test. Since that study, the beneficial mnemonic effects of intracranial infusions or systemic ghrelin treatment have been repeatedly replicated (Carlini et al., 2004, 2007, 2008, 2010a,b; Diano et al., 2006; Atcha et al., 2009; Tóth et al., 2010; Chen et al., 2011; Chen, 2012).

Importantly, beyond its effects on the circadian system and hippocampal dependent memory, ghrelin may also have potential for preventing or treating neurodegenerative disease.

Neurodegenerative disorders often display coexisting metabolic dysfunction, and there are several converging lines of evidence linking metabolic syndromes with an increased risk of developing AD (Naderali et al., 2009; Kapogiannis and Mattson, 2011; Cai et al., 2012). For example, there is a growing literature suggesting that insulin deficiency and insulin resistance act as mediators of AD-type neurodegeneration. This has led some to refer to AD as “type 3 diabetes”, a form of diabetes that selectively involves the brain (Steen et al., 2005; de la Monet and Wands, 2008). Because ghrelin has been shown to modulate insulin sensitivity (Chen et al., 2010), as well as several other metabolic and mnemonic effects, ghrelin may be a potential candidate molecule responsible for the relationship between metabolic and cognitive dysfunction. It is possible that disruption of the normal modulation of ghrelin secretion may contribute to the metabolic changes associated with AD.

Indeed, there is increasing evidence suggesting an association between ghrelin and AD pathology (Gahete et al., 2011). The first line of evidence is that involuntary weight loss and nutritional deficiencies are common in individuals diagnosed with AD, as well as being associated with cognitive impairment in non-demented elderly (Inelmen et al., 2009; Theodoropoulou et al., 2012).

**Figure 2:**
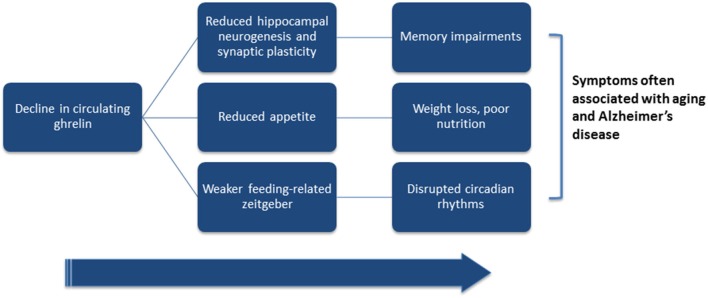
**Hypothesized link between ghrelin and symptoms often associated with aging and Alzheimer’s disease**. Ghrelin has been shown to directly affect hippocampal plasticity and neurogenesis, hunger-levels, and circadian processes. Both aging and Alzheimer’s disease (AD) are associated with lower levels of circulating ghrelin. Low ghrelin levels may result in reduced hippocampal plasticity and neurogenesis, and contribute to the cognitive deficits associated with old-age and AD. Lower circulating levels of ghrelin may also reduce hunger levels, and may partially underlie the weight loss associated with older age and AD. Finally, if ghrelin acts to enhance feeding-related zeitgebers, then reductions in circulating ghrelin could dampen food-entrained oscillators and disrupt circadian rhythmicity.

Importantly, weight loss may precede the memory loss associated with dementia (Buchman et al., 2005; Stewart et al., 2005; Johnson et al., 2006; Knopman et al., 2007). Because ghrelin is an important regulator of appetite, the age-related weight loss is in agreement with the finding of an age-related decline of plasma ghrelin concentrations as well as the age-related decline in growth-hormone releasing effect of ghrelin (Figure 2; Rigamonti et al., 2002; Broglio et al., 2003). AD patients compared with age-matched controls, also show a reduction in local ghrelin production in the brain (Gahete et al., 2010). Because weight loss appears to precede cognitive impairment in patients with AD (Knopman et al., 2007), metabolic changes could be targets for early detection and prevention of cognitive decline.

Although the human data examining the relationship between ghrelin and AD pathology is promising, there have been some mixed results. For example, one study reported that ghrelin levels do not vary in the cerebrospinal fluid of AD patients compared with age-matched controls (Proto et al., 2006) and another reported that ghrelin levels were negatively correlated with several cognitive domains, including verbal memory and working memory (Spitznagel et al., 2010).

The second line of evidence in support of a role of ghrelin in the development of AD comes from animal models. The first demonstration of a direct effect of ghrelin on AD-like alterations was in an AD mouse model (SAMP8), which develops an age-related increase in β-amyloid. In this model, ghrelin treatment improved retention on the T-maze foot shock avoidance task (Diano et al., 2006). Another mouse model, generated by intrahippocampal injection of oligometric forms of the Aβ peptide, demonstrated that systemic injection of ghrelin rescued performance on two behavioral paradigms (Y-maze and passive avoidance tasks), as well as attenuated AD-associated neuropathological abnormalities, possibly by inhibiting microgliosis and protecting neuronal integrity (Moon et al., 2014). Furthermore, neurons treated with ghrelin for 1 h show decreased tau hyperphosphorylation (Chen et al., 2010).

Although the evidence from human patients and from animal models is minimal and mixed, it is possible that an age-related decline in ghrelin may contribute to disruptions in age-related circadian disruption, weight loss, cognitive decline, and reductions in hippocampal neurogenesis (Figure 2). Because of this, well-timed supplements of ghrelin may have potential benefits for synchronizing an uncoupled circadian system, promoting hippocampal plasticity and neurogenesis, benefitting memory, and improving appetite and thus possibly nutrition, and may even help reduce pathology in AD patients.

Much more research needs to be done before drawing any conclusions, but taking a systems level approach that considers overall health hints at a potentially powerful role of ghrelin.

## Conclusions

It has been over a 100 years since AD was first identified by German psychiatrist and neuropathologist Aloysius Alzheimer, and yet we remain without effective treatments or a cure for this devastating disease. AD and other dementias are arguably the greatest global public health challenge of the 21st century. It is imperative that research go towards understanding the underlying disease processes at every level of analysis from genetics and molecular processes to systems and behavior; however, here it was proposed that there is great potential for identifying inexpensive and effective interventions by studying AD from a systems level approach that considers synchrony in the circadian system.

Aging is the greatest risk factor for developing AD. If we think about AD and other age-related neurodegenerative diseases as taking 60–70 years to develop, then it seems reasonable to think that there are ways to slow the development even further. Cell structures within tissues maintain a continuous synthesis and degradation of worn-out proteins that is integral to normal function. This process slows during aging, and the oxidative damage to proteins and protein misfolding lead to the accumulation of altered and abnormal proteins, which may contribute to neurodegenerative disease (Martinez-Vicente et al., 2005; Martinez-Vicente and Cuervo, 2007; Rattan, 2010). By promoting overall health, we may be able to maintain our natural defenses and cellular health.

Studying circadian rhythm disruption in AD holds great promise for inexpensive interventions. We know that harmonious interaction of internal and environmental rhythms ensures that physiological processes occur at an optimal time, and that maintaining this synchrony is best for longevity. If uncoupled circadian rhythms exacerbate symptoms or contribute to disease progression, then we may be able to draw from the food-entrainment literature to identify ways to synchronize peripheral rhythms with the SCN in elderly at risk for AD.

Future research should look for the earliest cues that internal rhythms are becoming uncoupled. A combination of light therapy, scheduled mealtimes, and other interventions, such as appropriately timed ghrelin administration, may have the potential to maintain circadian organization and synchrony throughout the body, and slow down the progression of disease. Delaying the onset of symptoms can potentially reduce the impact these devastating diseases have on the patients, their families, and society.

## Conflict of interest statement

The author declares that the research was conducted in the absence of any commercial or financial relationships that could be construed as a potential conflict of interest.
